# Diagnostic Validation and Feasibility of a Non-invasive Haemoglobin Screening Device (EzeCheck) for 'Anaemia Mukt Bharat' in India

**DOI:** 10.7759/cureus.52877

**Published:** 2024-01-24

**Authors:** Krushna Chandra Sahoo, Abhinav Sinha, Rakesh Kumar Sahoo, S. Shradha Suman, Debdutta Bhattacharya, Sanghamitra Pati

**Affiliations:** 1 Public Health, Health Technology Assessment in India, Indian Council of Medical Research (ICMR) - Regional Medical Research Centre (RMRC), Bhubaneswar, IND; 2 Microbiology, Health Technology Assessment in India, Indian Council of Medical Research (ICMR) - Regional Medical Research Centre (RMRC), Bhubaneswar, IND

**Keywords:** india, non-invasive, haemoglobin, point-of-care testing, anaemia

## Abstract

Anaemia remains a major public health issue in India despite several efforts. It is crucial to introduce technology-based innovations for the mass screening and early diagnosis of anaemia. Traditional anaemia screening requires drawing blood and laboratory analysis and can be logistically expensive in resource-constrained settings. A non-invasive haemoglobin test for mass screening in such settings is vital which can quickly and efficiently screen large populations. This study validated the haemoglobin estimation between the invasive haematology analyzer and the non-invasive EzeCheck (EzeRx Health Tech Pvt. Ltd., Bhubaneswar, Odisha, India) in the community setting. We conducted a cross-sectional study among 416 urban slum members in Bhubaneswar, India. We used inter-rater reliability (kappa statistic) of haemoglobin estimation between the haematology analyzer and EzeCheck devices. The finding showed a moderate agreement between both devices (kappa=0.4221). Between both devices, 91.59% of the results were with +/-1.5 difference; 43.51%, no difference; 33.65%, less than one difference; and 14.42%, +/-1 to +/-1.5 difference of haemoglobin estimation. There was no significant difference in overall anaemia status estimates between the devices. Mass screening in schools and communities with non-invasive haemoglobin tests can help identify anaemic people for early diagnosis and bring patients for timely treatment, which can be used in remote areas to support 'Anaemia Mukt Bharat'.

## Introduction

In India, anaemia is a significant public health issue, especially among women and children. In the most recent survey conducted in India, the National Family Health Survey (NFHS-5 hereafter), 2019-2021, the prevalence of anaemia increased dramatically across all age groups, with the greatest increase from NFHS-4 (2015-2016) occurring among children aged six months to five years, from 59% to 67%. In NFHS-4, 53.1% of the women aged 15-49 years were anaemic (11 g/dL), whereas, in NFHS-5, this percentage has increased to 57.1%. Similarly, in NFHS-4, 54.1% of the adolescent girls aged 15-19 years were anaemic, whereas, in NFHS-5, the prevalence increased to 59.1% [1]. Similarly, 29.2% of the adolescent boys aged 15-19 years were anaemic (13 g/dL) during NFHS-4 whereas 31.1% in NFHS-5 [2].

The Indian government has launched several anti-anaemia initiatives over the years, including the National Iron Plus Initiative (NIPI) [3], which provides iron and folic acid supplements to pregnant and lactating women and children. The 'Anaemia Mukt Bharat' (AMB) strategy or 'Anaemia-Free India' was launched in 2018 to reduce anaemia in vulnerable age groups especially women, children, and adolescents through a life cycle approach that includes preventive and curative mechanisms [3]. The significance of community anaemia screening lies in its ability to detect and address anaemia at an early stage, particularly in vulnerable populations, improving overall health outcomes and reducing the burden of this widespread condition.

Traditional community anaemia screening technology in India has typically involved invasive methods, primarily blood tests for haemoglobin (Hb) levels conducted in healthcare facilities or camps, which can be logistically challenging and resource-intensive [4]. The invasive methods of anaemia screening face several obstacles, most of which stem from the country's vast and diverse population, limited healthcare infrastructure, and socioeconomic disparities [5]. Accessibility is the greatest obstacle; many remote and underserved regions lack the necessary healthcare facilities and trained personnel to effectively administer invasive blood tests [6]. In addition, laboratory-based screenings are extremely expensive and could be time-consuming as well, which can be a barrier for individuals and the healthcare system. Certain segments of the population may be reluctant to participate in invasive screenings due to cultural influences and a fear of needles. In addition, the disposal of medical waste and the maintenance of sterile conditions present logistical challenges [7]. Transitioning to non-invasive or point-of-care methods for anaemia screening could be a more practical and inclusive approach to address this widespread health issue in the context of India's enormous healthcare burden. Therefore, community anaemia screening in India requires a low-cost, non-invasive technology that provides a quick and easy way to assess Hb status at the community level without the use of sophisticated equipment or invasive procedures [8].

Screening with a non-invasive Hb device can be a valuable tool for mass or community screening, especially for children and women because it is accessible, is cost-effective, is comfortable, provides quick results, is potable, generates zero biomedical waste, and has the potential to efficiently screen large populations and follow up on a real-time basis [9]. However, to ensure accurate results, the device should also have a high level of sensitivity and specificity. In addition, the developers could focus on the development of algorithms or software that can interpret the data collected by the device and provide actionable insights to healthcare professionals and patients. Moreover, the device should conduct real-time follow-ups and can be utilized in remote areas to support the AMB mission. The significance of a non-invasive Hb test for the mass screening of anaemia in low-resource settings cannot be overstated as it can be used for screening large populations quickly and efficiently. Therefore, this study validated the Hb estimation between the invasive haematology analyzer and the non-invasive EzeCheck (EzeRx Health Tech Pvt. Ltd., Bhubaneswar, Odisha, India) in a community setting.

## Materials and methods

Characteristics, testing algorithm, and principle of EzeCheck

**Figure 1:**
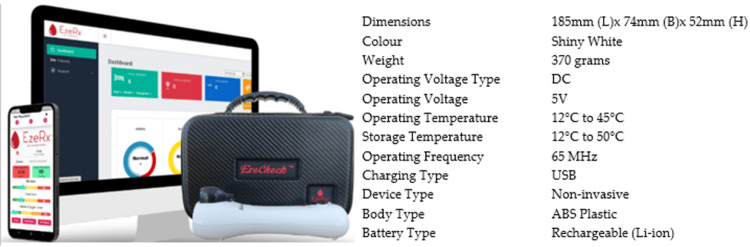
EzeCheck device specification.

EzeCheck is a non-invasive, portable screening device that can detect blood Hb in 10 seconds and does not require the user to give blood (https://ezerx.in/ezecheck). A cool white light-emitting diode (LED) light on the ring fingertip can collect data and send it to a mobile-based application for the healthcare provider who has been given the duty of screening the population. The software generates results in a few seconds using artificial intelligence (AI) and machine learning (ML) techniques based on the measurement of the spectroscopic signal. The devices often report Hb levels in grams per deciliter (g/dL). The units used are consistent with the standard units to ensure compatibility and ease of interpretation by healthcare professionals. All these reports, as well as demographic and patient data, can be transferred to a central database. Figure 1 depicts the detailed device specification.

**Figure 2:**
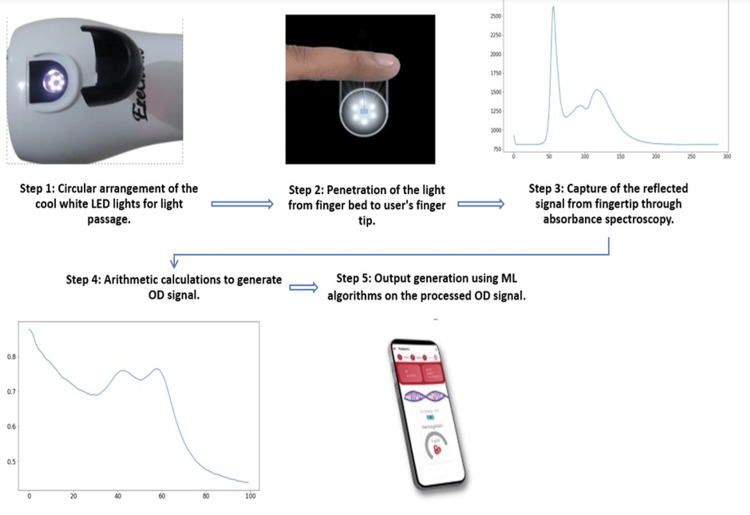
Explanatory diagram illustrating the measurement of the haemoglobin level in the sample.

The testing process is working on five major steps. In step 1, commencing with the device's mode of work, the EzeCheck has a distinguished area, known as the 'finger bed'. This region is marked on the device as a spherical structure which serves as the designated resting place for the subject's fingertip. Within this sphere, a cadre of six cool white LED lights has been positioned at precise 60-degree intervals, collectively forming a comprehensive 360-degree circle (6×60 degrees). In step 2, the luminous beam of light generated by these LEDs traverses the upper dermal layer of the fingertip, and upon their interaction with the body's biological substrate, it produces reflection. In step 3, the reflected light signal is then captured through the device using absorbance spectroscopy, within the visible spectrum range of 300-750 nm. In step 4, a simple arithmetic calculation is performed to process the received signal and convert it into an optical density (OD) signal for future processing: formula: log10 (signal-dark/reference-dark), where signal is the received reflected signal from the fingertip, dark is the signal reference with light intensity zero (no light condition), and reference is the spectrum obtained before the saturation level of a spectrometer. In step 5, this processed OD signal then undergoes certain ML algorithms for generating the Hb output, which is then displayed in the mobile app. Figure 2 depicts an explanatory diagram illustrating how the Hb level in the sample is measured (https://ezerx.in/ezecheck).

In order to determine the accuracy and efficacy of a non-invasive technology, it is necessary to use comparable standards. Keeping this in mind, the unit of measurement in both cases (invasive and non-invasive) was g/dL. According to information provided by the manufacturer of EzeCheck, in order to express the non-invasive output in g/dL, EzeCheck underwent the following series of logical and arithmetic expressions during its development: Initially, numerous spectroscopic signals were collected using EzeCheck simultaneously for individuals undergoing routine Hb tests using the conventional invasive technique, within a one-minute time interval. Following the collection of these training data sets ranging from extremely low to high Hb levels, a model comprised of both invasive and non-invasive data was constructed. The Hb values were generated using advanced algorithms based on the collected signals and classified into three categories: low, normal, and high.

The process was repeated for large data sets over a year, including several critical and non-critical cases, as well as patients with various anomalies, sex, groups, skin tones, and thicknesses [9]. A matrix was developed based on the built model (consisting of large data sets), in which g/dL was identified as the standard for the values generated by the EzeCheck device since a strong correlation was observed between the Hb reports generated by the invasive method and those generated by the non-invasive EzeCheck device [9]. The collected spectroscopic signal patterns with respect to the Hb values generated by the EzeCheck device were then cross-verified with the invasive reports to determine the potency of the non-invasive method for determining Hb values in terms of g/dL. Although the non-invasive technologies provide the Hb values in relative units, the algorithm and the matrix generated by collecting a huge number of data sets enabled the manufacturer to express the non-invasive Hb values in terms of g/dL.

The EzeCheck device operates based on absorption spectroscopy. When the subject places his or her finger on the device, the LED light present in the finger bed region of the device begins to attenuate from the reference spectrum to the dark spectrum, and each intensity collects spectral signals from the specified range of 300-750 nm (visible spectrum range). An output is generated in the form of an absorbed signal at nearly 10 different intensities, within the range of 300-750 nm, from which a stipulated part of the signal (450-700 nm) is taken further for processing. Out of the 10 signals present at different intensities, a single specific suitable signal (specifically within the spectral range of 530-631 nm) is selected based on the various mathematical permutation combinations of different wavelength points within the specified wavelength range of 530-631 nm. After identifying the appropriate signal along with the first signal point (at 450 nm) and the last signal point (at 700 nm), the amplitude points of the signals are utilized to generate the Hb output using the ML algorithm (multi-dimensional linear regression). The received output is sent to the mobile application for display to the subject.

Feasibility of EzeCheck use

Any laboratory or medical test that aids in determining histopathology, biomarkers, diagnosis, and prognosis, typically necessitates the drawing of blood samples or the operation of sample processing machines by professional doctors or trained laboratory personnel. EzeCheck, on the other hand, is a simple and easy-to-use screening device that allows quick-determining Hb results if the person is familiar with using an Android mobile phone. 

Study design, sample size, and sampling

We carried out a cross-sectional study among the urban slum community members in Bhubaneswar, Odisha, India. We calculated the sample size based on the guidelines of the minimum sample size requirements for Cohen's kappa [10]. Cohen's kappa coefficient is a test statistic that measures the level of concordance between two distinct evaluations of a response variable. To simplify all calculations, alpha and power are usually set at 0.05 and 80%, respectively, and with an effect size of K1=0.0 vs K2=0.2, the two-category yields a minimum sample size of 194 for each device. Alpha (α) is the significance level used in hypothesis testing, indicating the likelihood of type I errors. A lower alpha (e.g., 0.05) signifies stricter significance. Power (1-β) reflects the test's sensitivity to detect true agreement, with higher values (e.g., 0.80) indicating better detection ability, reducing type II errors. These parameters are essential for evaluating inter-rater reliability effectively [11]. Hence, we included a total of 416 individuals in this validation study. We selected a total of seven slums and one government school residing in the vicinity of the slum setting from the north zone of Bhubaneswar City. From each site, a minimum of 50 individuals participated in the study.

Cohen's kappa is a valuable measure for assessing agreement among raters, considering chance agreement. It helps researchers evaluate reliability and validity. The kappa value ranges from -1 to 1, with 1 indicating perfect agreement and 0 suggesting agreement by chance. The interpretation of kappa results is as follows: values ≤0 indicate no agreement, 0.01-0.20 suggest none to slight agreement, 0.21-0.40 represent fair agreement, 0.41-0.60 signify moderate agreement, 0.61-0.80 indicate substantial agreement, and 0.81-1.00 reflect almost perfect agreement [11].

Data collection procedure

The Hb concentration was tested using automated haematology analyzers at a National Accreditation Board for Testing and Calibration Laboratories (NABL)-accredited laboratory. The laboratory staff collected venous blood samples from all individuals at the field site using a sterile needle and syringe. The blood sample was then transferred into a collection container suitable for use with the haematology analyzer following the manufacturer's recommendations for the anticoagulant and the amount of blood required. In the haematology analyzer, the test tube or collection container was then placed. Then, the analyzer was switched on, and the appropriate Hb test was selected. We then waited for the analyzer to finish the analysis, which could take a few minutes. The Hb level displayed on the analyzer screen was noted. Hb measurement was reported in g/dL. Appropriate safety procedures to dispose of the blood sample and any waste materials were followed.

To obtain accurate result values, we used the following steps during the test in EzeCheck. First, we downloaded and installed the EzeCheck app from the Play Store on the Android mobile phone and then turned 'ON' the mobile data, location, and Bluetooth on the mobile phone. We logged in to the EzeCheck app using the username and password provided and switched on the EzeCheck device by pressing the black button. Then, we clicked on 'add patient' option and filled in the basic details of the patient followed by pressing the 'submit patient' button.

Before conducting the test, clean the left-hand ring finger tip using a cloth. Gently place the finger on the device's finger bed area. Connect the EzeCheck device to your mobile's EzeCheck app, maintain a stable hand posture until the test reaches 100% completion, and receive results within 60 seconds. The test result will be displayed on the mobile screen. For a retest, click the 'Redo' icon. To view and save the report in PDF format, click 'Download'. To perform the test for another patient, click the Plus icon.

Data management and analysis

We used inter-rater reliability (kappa statistic) of Hb estimation between the haematology analyzer (invasive) and EzeCheck (non-invasive) devices. The objective was to determine the degree to which responses at time 2 are consistent with responses at time 1. Inter-rater agreement is typically a measure of the degree to which different raters using the same scale, instrument, classification, or procedure to evaluate the same objects agree with one another (or subjects). The intra-rater agreement, on the other hand, is also known as test-retest agreement, which is a measure of the agreement by the same rater, who may be employing the same scale, instrument, classification, or procedure to assess the same objects (or subjects) at two different times. The range of kappa's coefficient is between -1 and 1, with kappa equal to -1 representing perfect disagreement and kappa equal to 1 representing perfect agreement.

The frequency and percentage of variation of results between the haematology analyzer and EzeCheck devices are presented with a range of differences such as 0, less than 1, 1-1.9, 2-2.9, 3-3.9, and 4 and above in g/dL. We presented the anaemia classification as followed by the National Family Health Survey [12] and the World Health Organization (WHO) classification of anaemia according to age and severity [13].

Ethical considerations

Ethical clearance was obtained from the Institutional Ethical Committee of Kalinga Institute of Medical Sciences, Bhubaneswar, India (approval number: KIIT/KIMS/IEC/543/2021). We obtained consent from all the participants and assent from those below 18 years of age before blood sample collection including parental consent. All necessary measures were taken to ensure the privacy and confidentiality of the data.

## Results

A total of 416 urban slum community members participated in the study, among them 60% (n=248) were females and 40% (n=168) were males. A total of 73% were aged 15 years and above. Among the married women, 12 were currently pregnant. The detailed participants' characteristics are presented in Table 1.

**Table 1:** Participants' characteristics.

Characteristics	Total (N=416)		Male (N=168)		Female (N=248)	
	n	%	n	%	n	%
Mean age (range), SD	31 (6-75), 17.85					
Age (in years)						
5-11	52	12.5	30	17.9	22	8.9
12-14	59	14.2	29	17.2	30	12.1
15 and above	305	73.3	109	64.9	196	79
Caste/religion						
General	64	15.4	24	14.3	40	16.1
Other backward class	61	14.7	23	14	38	15.3
Scheduled caste	134	32.2	59	35.1	75	30.2
Scheduled tribes	147	35.3	61	36.3	86	34.7
Minority	10	2.4	1	0.6	9	3.6
Marital status						
Unmarried	168	40.4	88	52.4	80	32.2
Married	224	53.8	78	46.4	146	58.9
Widow/widower	24	5.8	2	1.2	22	8.9
Currently pregnant	NA	NA	NA	NA	12	8.2
Education						
No formal schooling	77	18.5	22	13.1	55	22.2
Primary school (first to fifth)	94	22.6	48	28.6	46	15.5
Secondary (sixth and seventh)	95	22.8	37	22	58	23.4
High school (eighth to 12th)	111	26.7	40	23.8	71	28.6
Graduation and above	39	9.4	21	12.5	18	7.3
Occupation						
Student	154	37	75	44.6	79	31.8
Homemaker	115	27.6	NA	NA	115	46.4
Shopkeeper	15	3.6	11	6.5	4	1.6
Private jobs	44	10.6	24	14.3	20	8.1
Daily labour	88	21.2	58	34.5	30	12.1

The inter-rater reliability (kappa statistic) of Hb estimation in g/dL between the haematology analyzer (invasive) and EzeCheck (non-invasive) devices is presented in Table 2. The finding showed a moderate agreement between both devices (agreement=43.51% and kappa=0.4221).

**Table 2:** Inter-rater reliability (kappa statistic) of haemoglobin estimation (g/dL) between the haematology analyzer (invasive) and EzeCheck (non-invasive) devices.

Agreement	Kappa	Standard error	Z	Prob>Z	Inference
43.51%	0.4221	0.0074	57.17	0.0000	Moderate agreement

**Figure 3:**
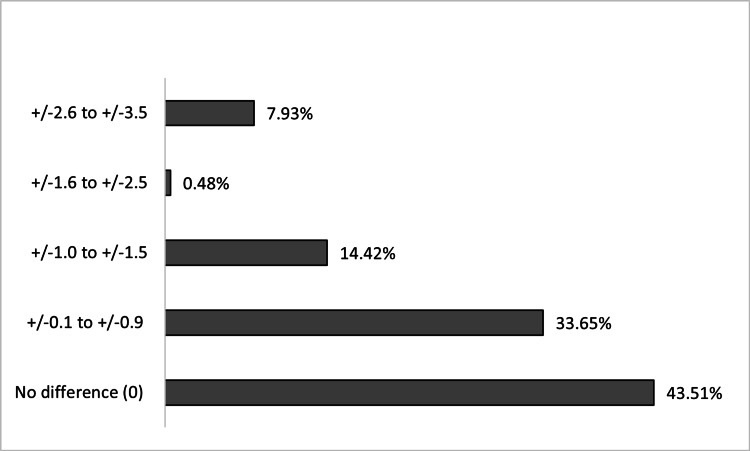
Variation of results between the haematology analyzer (invasive) and EzeCheck (non-invasive) devices.

Between both devices, 91.59% of results are with +/-1.5 difference (43.51% no difference, 33.65% less than one difference, and 14.42% with +/-1 to +/-1.5 difference) of Hb estimation (g/dL) (Figure 3). 

**Figure 4:**
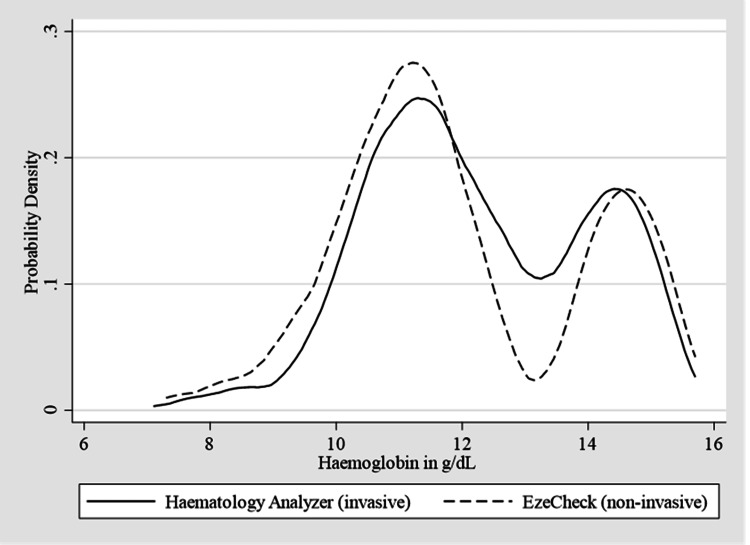
Comparative measurement of haemoglobin (g/dL) in the haematology analyzer and EzeCheck.

This study compared the accuracy of Hb levels (g/dL) measured using non-invasive and invasive methods. The EzeCheck device (non-invasive) recorded Hb levels of 11.98±1.9 g/dL (mean±standard deviation (SD)) with a range of 7.3-15.7, while the mean Hb level obtained from the collected blood in the haematology analyzer (invasive) was 12.24±1.7 g/dL with a range of 7.1-15.7. Therefore, the mean difference of SD was 0.18 g/dL. Figure 4 displays the density distributions of the confidence ratings between Hb (g/dL) in the haematology analyzer and EzeCheck. A density distribution, which is frequently represented as a probability density function, illustrates how a variable's values are distributed or concentrated across its possible range.

The comparison with various Hb estimation methods is shown in Table 3.

**Table 3:** Comparative analysis of haemoglobin estimation in invasive (haematology analyzer) and non-invasive (EzeCheck) devices and anaemia status among men, women, and children according to NFHS standard. NFHS: National Family Health Survey

Population group	Diagnostic tool results	Anaemia status				p-value (chi-squared/Fisher)
		Severe (n)	Moderate (n)	Mild (n)	Normal (n)	
Men, 15 years and above (N=109)	Haematology analyzer	1	11	7	90	0.063
	EzeCheck	1	23	2	83	
	Difference	0	12	5	7	
Women, 15 years and above (N=184)	Haematology analyzer	0	19	120	45	0.007
	EzeCheck	0	27	141	16	
	Difference	0	8	21	29	
Pregnant women (N=12)	Haematology analyzer	0	0	1	11	0.613
	EzeCheck	0	1	3	8	
	Difference	0	1	2	3	
Children, 5-11 years (N=52)	Haematology analyzer	0	10	6	36	0.587
	EzeCheck	0	16	6	30	
	Difference	0	6	0	6	
Children, 12-14 years (N=59)	Haematology analyzer	0	11	11	37	0.099
	EzeCheck	2	19	14	24	
	Difference	2	8	3	13	

## Discussion

Anaemia screening in India is a critical public health initiative due to the high prevalence of anaemia in the country, especially among women and children. Despite several efforts, anaemia remains a significant public health challenge in India. It is a critical to combat anaemia and improve the health and well-being of the Indian population, particularly vulnerable groups like women and children. We observed a moderate agreement between the two devices, i.e., non-invasive EzeCheck and invasive haematology analyzer, which is the major strength of the device. The comparison of various commonly used Hb estimation methods is presented in Table 4.

**Table 4:** Comparison with available haemoglobin estimation methods.

Measuring parameters	EzeCheck	Sahli's method	One Drop kit	Haematology analyzer
Blood sample quantity	No blood	20-50 uL	8-10 uL	200-500 uL
Separate container	Not required	Required	Not required	Required
Consumables	Not required	Required	Required	Required
Biomedical waste	No	Yes	Yes	Yes
Lab technician	Not required	Required	Required	Required
Time for result generation	60 sec	20-30 min	60 sec	Three hours including sample transportation
Community-level screening	Yes	Time-consuming	Yes	Not possible
Parent consent in case of age<18 to take blood	Not required	Required	Required	Required
Portability	Yes	Not possible	Yes	Not possible
Immediate data transfer to the next level for medication/follow-up	Available	Not available	Not available	Not available
Operation by any non-technical person	Yes	Not possible	Not possible	Not possible
Any dedicated space or room	Not required	Dedicated lab	Anywhere	Dedicated lab
Unit test cost (in Rs.)	<5	>8	>15	>50
Accuracy	>90%	>80%	>90%	>95%
Training for diagnosis	One hour only to any semi-skilled manpower	>1 month to lab technician	>1 day to skilled manpower	>1 month to lab technician

The biggest advantage of using a non-invasive Hb screening method is that it is simple and easy to use, can be performed in a non-medical setting, and is easy to operate by any semi-skilled manpower. It is a simple and convenient method for screening large populations. The traditional invasive methods require expensive laboratory equipment, consumables, and trained medical personnel which bears high cost (approximately 40-60 Indian rupee (INR) per test), whereas non-invasive Hb screening for anaemia or other blood disorders in large populations is low-cost (approximately 5-7 INR per test, including device cost (EzeCheck)). Moreover, the latter does not generate biomedical waste.

Non-invasive tests provide rapid results, often within seconds or minutes. This quick turnaround time enables healthcare providers to screen a large number of individuals efficiently, making it ideal for mass screenings and outreach programmes [14]. Drawing blood can be uncomfortable and even intimidating for some individuals, particularly children. Non-invasive tests eliminate the need for needles and blood samples, making the screening process more tolerable and less anxiety-inducing [15]. In resource-limited settings, where proper sterilization and disposal of medical equipment may be challenging, non-invasive tests mitigate the risk of infection transmission associated with needle-based blood draws [16]. Non-invasive tests can be performed more frequently if necessary, allowing for better monitoring of anaemia prevalence and treatment efficacy over time.

The quicker results through non-invasive methods allow for immediate intervention or referral to medical professionals, if necessary [17]. Furthermore, the device can do follow-up longitudinally. Non-invasive Hb screening methods have the potential to screen large populations quickly and efficiently, which is especially useful for mass screening programmes in low- and middle-income countries (LMICs) such as India or during disaster relief situations [18]. Moreover, it also doesn't require any consent/assent from parents for blood, which needs to be taken from <18-year-old children.

The non-invasive Hb tests, such as those based on spectrophotometry or photoplethysmography, do not require specialized training [19]. They can be performed by healthcare workers with minimal training, making them accessible even in remote or underserved areas. Thus, non-invasive tests offer a cost-effective alternative, reducing the financial burden on healthcare systems and patients, and have a potential for wider reach [20]. A smartphone app for the non-invasive detection of anaemia using patient-supplied photographs detected anaemia (Hb levels 12.5 g/dL) with an accuracy of 2.4 g/dL and a sensitivity of 97% (95% CI, 89-100%) when compared to complete blood count (CBC) Hb levels (n=100 subjects) [21]. A recent review article highlights the potential of non-invasive systems for the monitoring and early detection of anaemia, which is a significant problem [22].

India has one of the highest rates of anaemia in the world. In India, anaemia screening is often conducted in healthcare facilities, including government health centres and private clinics. Mobile health camps are also set up in remote areas to reach underserved populations and among schoolchildren [23]. These programmes aim to identify anaemic children and provide them with the necessary interventions, such as iron and folic acid supplementation. However, these programmes are dependent on the use of skilled manpower to test anaemia by drawing blood which may change through the use of non-invasive methods. Nonetheless, public awareness and education campaigns are essential to promote the importance of proper nutrition and early detection of anaemia [24]. These campaigns often target pregnant women, new mothers, and caregivers of young children.

Anaemia, if detected early through non-invasive testing, can be managed more effectively, potentially preventing more severe health complications down the line. Non-invasive tests are well-suited for community-based healthcare initiatives and mobile clinics. They enable healthcare workers to reach marginalized populations who may not have easy access to healthcare facilities. By providing a simple, non-invasive screening method, healthcare systems can work toward achieving greater health equity, ensuring that all individuals, regardless of their location or socioeconomic status, have access to essential anaemia screening services. Non-invasive Hb tests are a game-changer in the mass screening of anaemia, particularly in low-resource settings. They offer a practical, cost-effective, and patient-friendly approach to identifying anaemia, ultimately contributing to better health outcomes and improved quality of life for populations in need [20].

The health sub-centre/primary health centres/Ayushman Bharat Health and Wellness Centres serve as the primary point of contact between the healthcare system and the community in India. The success of national health programmes relies heavily on these centres providing quality services. To address the issue of anaemia, a device can be implemented at these centres for mass screening by frontline workers such as accredited social health activist (ASHA) and auxiliary nurse midwife (ANM). The Ayushman Bharat 'School Health Program' aims to promote health and disease prevention in government and government-aided schools. Regular screening of schoolchildren for anaemia without blood samples is recommended. The Odisha government has identified women's empowerment as a key development initiative and should conduct regular anaemia screenings for adolescent females. Anganwadi centres, which are rural child day care centres, provide basic healthcare activities such as contraception counseling, nutrition education, and preschool activities. The device can be used at the Anganwadi level for the continuous screening of rural children.

EzeCheck's main strength is that it captures primary patient information through the Android app and connects to the mobile device via Bluetooth. The solutions are affordable and accessible to the community. It provides immediate results and remote access to data for a rapid treatment plan. It features Internet of Things (IoT)-enabled offline storage capabilities and historical data trends that aid in real-time disease monitoring and follow-up. Operating the EzeCheck app is user-friendly and straightforward, requiring only basic reading and writing skills in English. Anyone familiar with using an Android smartphone can handle it with ease. Despite being stored on a cloud server, the data maintains a high level of security and confidentiality.

The device can also function offline when network connectivity is unavailable. It prevents the loss of 5-10 ml of blood and renders the procedure infection-free and painless. Since the entire process generates instant results, a great number of man-days/man-hours are saved, which incurs an opportunity cost. Furthermore, as a non-invasive technique, it generates no medical waste. It can be used on any population, including diabetic patients, because it never causes infection or cross-infection. The primary and secondary patient data collected in real-time from the field by healthcare professionals can be monitored via a centralised, interactive dashboard for device management in specific territories. In addition, geotagging capabilities will be useful for gaining insight into a particular geography. Each device can be monitored for the number of tests conducted per day, allowing policymakers to determine the next steps based on the collected data.

The EzeCheck non-invasive Hb device has several limitations, including being a screening device for individuals aged four years and above, precautionary use for those under four years old, and the need to avoid moisture, cut, or stain marks on the left-hand ring finger. The study on this device also has drawbacks, such as not comparing it to other non-invasive approaches and showing different results compared to a haematology analyzer. To improve the accuracy of the EzeCheck device, it is suggested to train and evaluate it using a diverse data set, calibrate and standardize the equipment regularly, and conduct rigorous clinical validation studies.

## Conclusions

Non-invasive Hb testing has the potential to be a valuable tool for mass screening in schools and communities. Non-invasive Hb tests can aid in the identification of people suffering from anaemia and other blood disorders, allowing for early intervention and treatment. Furthermore, the use of this device in mass screening programmes can assist in identifying groups of people who are at a higher risk of anaemia, such as those from low-income families or those with limited access to healthcare. This data can be used to create targeted interventions and programmes to combat these disparities. Overall, non-invasive Hb tests can be a valuable tool for mass screening in schools and communities, assisting in the identification of individuals affected by anaemia and other blood disorders and improving health outcomes and quality of life.
